# The Bulgarian Version of the Multidimensional Driving Style Inventory: Psychometric Properties

**DOI:** 10.3390/bs9120145

**Published:** 2019-12-08

**Authors:** Zornitsa Totkova, Rositsa Racheva

**Affiliations:** Department of Psychology, Institute for Population and Human Studies, Bulgarian Academy of Sciences. Acad. G. Bonchev St., Bl. 6, Fl. 5, 1113 Sofia, Bulgaria; z.totkova@iphs.eu

**Keywords:** driving style, assessment, instrument reliability, MDSI-BG, Bulgarian adaptation

## Abstract

Road safety is one of the main priorities for the European Union. Different strategies and policies strive to increase the level of road safety across Europe and although this level has increased in the last couple of years the number of injuries and fatalities resulting from traffic accidents is still very high. The multidimensional driving style inventory (MDSI) is a self-reported instrument for the assessment of a person’s habitual driving style and its connection to risky driving behaviour and involvement in different traffic accidents. The instrument was originally developed in Israel and there are several previous adaptations in different countries such as Argentina and Romania. The main objective of this study is to develop a valid and reliable version of the MDSI in Bulgaria. A study was conducted to evaluate the construction validity of the instrument and to test the validity of the factors in a Bulgarian sample (n = 456, male = 204; female = 252; average age = 37). Eight factors representing a specific driving style—dissociative, anxious, risky, angry, high-velocity, distress reduction, patient and careful and irrational—identified by previous versions of the MDSI are included in this study. The overall number of items in the inventory is 57 with Cronbach’s alpha = 0.78. The current study in Bulgaria confirmed the structural organization of the initial version of the inventory. The results of the conducted study supported the reliability and validity of the Bulgarian version of the MDSI. The possible implementation of the instrument for the development of different programs for preventions and interventions is discussed here within.

## 1. Introduction

In December 2018, the World Health Organization presented the Global status report on road safety which highlights that the number of annual road traffic deaths has reached 1.35 million. Road traffic injuries are now the eighth leading cause of death for people of all ages and the number one cause of death for children and young adults aged 5–29 years. Death rates in low-income countries are three times higher than in countries with high-income [1]. Although Bulgaria accepted a strategy for increasing the level of road safety almost 10 years ago, significant results have not been accomplished. In 2018, more than 8400 people were injured and 611 people died on Bulgarian roads. Contemporary scientific research in the field of transport psychology lags behind other countries inside and outside of the European Union. The need for such research in recent years has become more and more tangible, both because of the increased public intolerance to aggressive and risky driving and because of the very high number of injuries and fatalities in road accidents in the country.

**Figure 1:**
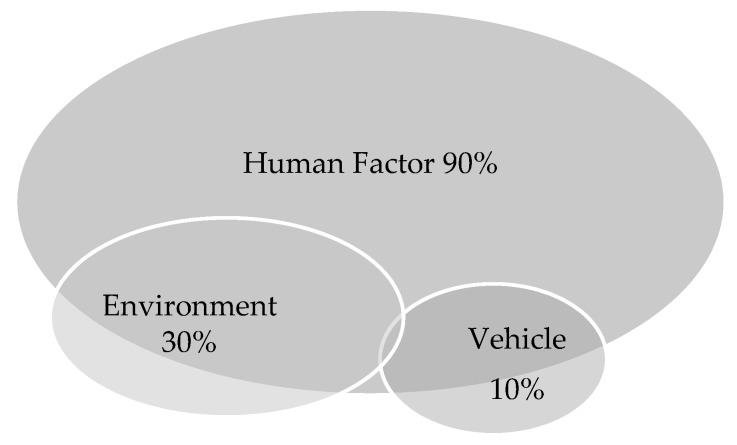
European Report on Accident Research and Safety Report (2017).

The interacting factors that lead up to an accident can be grouped into three categories: human factor, environment and vehicle (Figure 1).

An analysis of research on the topic shows that about 30% of emerging road accidents are due to environmental factors (slippery roads, poor visibility, weather conditions, etc.) and another 10% of road accidents are related to vehicles (flat tires, bad maintenance, etc.). It is also known that the human factor contributes significantly to 90–95% of road accidents [2].

Research on the role of the human factor in other countries has shown that driver behaviour should be seen as an action determined by various components: individual characteristics, knowledge, skills and driving habits, functional and mental state during driving and more. That is why the study of different driving components such as behaviour, skills and styles is crucial for understanding why the level of road safety in the country has not increased significantly despite all changes in the road legislation in Bulgaria.

### 1.1. Driving Styles

In recent years, scientists conducting academic and empirical studies on driving have begun to differentiate not only between certain driving behaviours and abilities, but also driving styles. The driving style is defined by different authors over the years. Here are some of the definitions: “Driving style concerns individual driving habits—that is, the way a driver chooses to drive” [3]; “Driving style concerns the way individuals choose to drive, or driving habits that have become established over a period of years” [4]; “Dynamic behaviour of a driver on the road” [5]; “Driving style is concerned with decision making aspects of driving, that is, the manner in which people choose to drive or driving habits that have developed over time” [6]; “An attitude, orientation and a way of thinking for daily driving” [7].

Based on these definitions and despite the differences between them, there are some aspects that most often determine the driving style. First, the style of driving is different for individuals or groups of individuals. Second: the driving style is the habitual way of driving, which is a relatively stable element of driving behaviour. Third: the driving style reflects the conscious choices the driver makes [8]. In general, the concept is that the driving style is the usual way of driving which is specific for a certain driver. The habitual driving pattern refers to the behaviour on the road that occurs constantly in different situations and includes both automated actions and consciously controlled behaviour by the driver. The most systematic viewpoint of driving style is derived from a multidimensional approach that revealed four broad dimensions [9]. The first dimension is defined as ‘reckless and careless driving’ and consists of two driving styles: risky style and high-velocity style. These styles are associated with sensation and thrill-seeking while driving and also with violations of the road safety norms. The second dimension is ‘anxious driving’ and includes anxious, dissociative and distress-reduction styles. These styles are more often characterized by a feeling of tension and pressure and inability to relax during driving. The third general dimension is ‘angry and hostile driving’ and includes the angry driving style. This style is associated with feelings of anger, irritation and impatience, and with acts of hostile and aggressive driving. The last dimension is the ‘patient and careful driving’ which includes two styles: careful and patient styles. These styles are characterized by patience, attention and tolerance while driving, as well as behaviour which is reasonable and healthy during driving [10,11].

### 1.2. The Multidimensional Driving Style Inventory

This concept is the basis on which the multidimensional driving style inventory (MDSI) was developed [9]. There are many different instruments for assessing the driving styles: Driving Style Questionnaire [7,12,13]; Driving behaviour questionnaire [14,15]; Driving Behaviour Inventory [16]. The multidimensional driving style inventory enables analysing driving on a comprehensive level including behaviours and habits related to driving in general by adapting items from some of the mentioned instruments as well as constructing original items [11]. The original version of the Inventory includes 44 items measuring the styles described above. There is much evidence supporting the advantage of this instrument. First of all, it defines and includes eight different factors that were primarily measured as separate concepts by previous instruments. Second, the MDSI determines positive driving behaviours that were mostly neglected in previous research because of focusing on the behaviours associated with risky driving. Third, the method was originally created in Israel in 2004, it was re-evaluated a decade later and was adopted in different cultural contexts in countries like Brazil, Argentina, Belgium, Netherland, Malaysia and Romania [10,11,17,18,19]. The original development of the instrument reported several indicators of the MDSI’s reliability and validity [9]. The angry, risky and high-velocity driving styles were positively and significantly associated with self-reports of vehicle crashes as well as with traffic offences, and the careful driving style was significantly related to fewer traffic crashes [10]. All this evidence and the listed qualities suggest a clear advantage for the use of MDSI as a reliable and valid instrument in traffic psychology research. On this basis, we consider that the MDSI is a valid and reliable instrument for the assessment of the driving styles, which are very significant elements of the driving process and the research in the field of traffic psychology.

### 1.3. Aim of the Present Research

It was mentioned that Bulgaria shows a significant delay in contemporary studies in the field of traffic psychology and Bulgarian driver’s behaviour. The analyses on this topic are mostly derived from different statistical reports of the Ministry of Interior on traffic rules violations, road accidents, etc. The policies concerning the road safety level in Bulgaria are mostly based on expert opinions and foreign policies rather than on scientific evidence. The first step to accomplish scientific results concerning driver behaviour in Bulgaria is to adopt and develop reliable and valid measurement instruments.

That is why the main objective of this study is to develop a valid and reliable Bulgarian version of the multidimensional driving style inventory (MDSI-BG) and to investigate its psychometric qualities.

## 2. Materials and Methods

### 2.1. Participants

Four hundred and fifty-six Bulgarian drivers from the general population participated in this study. Data collected from October 2018 until December 2018. The age of the participants ranged between 19 and 72 years (M = 37.00; SD = 9.07). According to gender, the sample includes 204 (45%) men and 252 (55%) women. In regard to occupation, the largest percentage of the participants (87%) are from the city of Sofia and the remaining participants (13%) are from various geographical areas in Bulgaria (Plovdiv, Varna, Pleven, Burgas, Haskovo, etc.). In regard to education, 79.4% of the participants possess a university degree and 20.6% completed high school. 

All of the participants have a driving license, 86.8% of them drive every day or at least 2–3 times per week, and 13.2% drive 2–3 times per month or even rarely. Of the surveyed participants, 69.5% have registered traffic violations in the past 3 years. Violations that are most often recorded include speeding, illegal parking, using a phone while driving, driving without a seat belt and driving after alcohol consumption. The sample also includes 6.1% of surveyed persons who have been deprived of a driving license due to different offences—most often for speeding, driving after alcohol consumption and involvement in road accidents. Participants who had caused a road accident account for 39%. Victims in a traffic accident caused by another driver account for 58.8% of the surveyed persons. Participants included in the study were not paid for their participation.

### 2.2. Measures

The Bulgarian version of the MDSI was developed from previous versions of the inventory, namely the original MDSI [9], the Argentinian version [10] and the Romanian version [11]. In order to maintain as much correspondence as possible between the Bulgarian version of the MDSI and these previous versions, the MDSI-BG included 44 items from the original instrument, 12 items from the Argentinian version and 7 items from the Romanian adaptation of the inventory. To adopt the instrument to the Bulgarian cultural context as much as possible some differences were made while the instrument was modified. For the purposes of the correct translation into Bulgarian and the development of a suitable method for the Bulgarian sample the following procedures were performed:

- Right and reverse translation in items formulation;
- Evaluation by experts (3 psychologists and 1 road safety expert);
- Evaluation consistency (approximately 90%).

All of the items are compared to their original versions and edited according to the inconsistencies so as to achieve the highest possible level of correspondence between the original items and their translation into Bulgarian.

Participants also reported their socio-demographic characteristics-age, gender, education and occupation. They were also asked to provide some information regarding their driving experience—years of driving, type of license, how often do they drive, what kind of violations they have committed in the last three years, caused traffic incidents, etc.

### 2.3. Procedure

An online version of the constructed instrument was prepared to facilitate the process of data collection. A link to the inventory was sent to email and social media contacts selected on a random basis (every third of the contact list). Participants were informed that the research is part of a scientific project financed by a program for supporting young scientists in Bulgaria. The response rate was more than 95%. The questionnaire was completed in 20 min on average. Data management and analysis were performed using SPSS 22. The statistical analysis involved two phases: (a) confirmatory factor analysis to verify the structure of the MDSI-BG and (b) reliability analysis (internal consistency analysis) of the resulting factor scales.

## 3. Results

### 3.1. Factor Analysis

The extended version of the Bulgarian version of MDSI was subjected to confirmatory factor analysis in order to verify the factor structure of the inventory (extraction: primary axis; rotation: Quartimax). The number of eight factors were preliminarily defined. The analysis revealed that these factors explained 52.29% of the total variance. Bartlett’s test for sphericity is used to check if the items are appropriate for the implementation of factor analyses (KMO = 0.848, Bartlett’s test = 14397.59, p < 0.001). The results from the factor analyses are presented in Table 1.

The first factor included nine items (factor weights ranging between 0.326 and 0.817) corresponding to the risky driving style and explained 8.25% of the variance. The second factor consisted of five items (factor weights ranging between 0.074 and 0.672) describing the irrational driving style and explained 7.86% of the variance. The third factor combined seven items (factor weights ranging between 0.420 and 0.848) defining the anxious driving style and explained 7.58% of the variance. The next factor was represented by six items (factor weights ranging between 0.382 and 0.729) corresponding to the high velocity driving style and explained 7.06% of the variance. The fifth factor consisted of eleven items (factor weights ranging between 0.350 and 0.632) describing the dissociative driving style where the explained variance was 7.00%. The sixth factor included eleven items (factor weight ranging between 0.265 and 0.685) and described the patient and careful driving style. This factor explained 5.95% of the variance. The next factor included seven items (factor weights ranging between 0.317 and 0.656) corresponding to the angry driving style and explained 4.76% of the variance. The last factor was represented by six items (factor weights ranging between 0.438 and 0.766) characterizing the distress reduction style and the explained variance was 3.79%. These results revealed the initial content of each one of the scales.

### 3.2. Reliability Analyses

To verify the reliability of the MDSI-BG, each scale was submitted to internal consistency analysis. The initial processing of the results demonstrated that some items did not show an acceptable item correlation with the correspondent scales. For the risky driving style scale, the item “Fix my hair/makeup while driving” had a low item-total correlation value and allowed an increase of the Cronbach’s alpha coefficient if deleted from α = 0.844 to α = 0.863. For the irrational driving style scale, the item that did not show an acceptable value was “Don‘t fasten my seatbelt during short trips of less than 10–15 min in the city”. It was deleted and the Cronbach’s alpha coefficient increased from α = 0.756 to α = 0.805. For the anxious driving style scale, the item “On a clear freeway, I usually drive at or a little below the speed limit” had a low factor loading and it was deleted, which led to an increase in the values of Cronbach’s alpha coefficient from α = 0.781 to α = 0.835. Regarding the dissociative driving style scale, two items had low item-total correlation values and allowed an increase of the Cronbach’s alpha coefficient when deleted from α = 0.728 to α = 0.767. These items were “Misjudge the speed of an oncoming vehicle when passing” and “I daydream to pass the time while driving”. There was one item in the patient and careful driving style scale that gave the opportunity to increase the Cronbach’s alpha coefficient. After deleting the “Always ready to react to unexpected manoeuvres by other drivers” item the values increased from α = 0.719 to α = 0.734. All other scales did not give the opportunity to make changes to increase the Cronbach’s alpha coefficient and to achieve more inter-correlated scales. For the high velocity driving style scale α = 0.741; for the patient and careful driving style scale α = 0.719; for the angry driving style scale α = 0.746; and for the distress reduction driving style scale α = 0.626. The results are presented in Table 2:

The results show that all but one of the eight factors (distress reduction) reached acceptable (> 0.70) Cronbach’s alpha coefficients. These results largely replicate the results presented in the previous studies for adapting the MDSI. 

On the basis of the conduction of different analyses, the obtained results and the comparison to the previous version of the MDSI, it can be claimed that the Bulgarian version of the multidimensional driving style inventory (MDSI-BG) demonstrates very good psychometric properties. The eight-factor structure of the instrument successfully combined all the positive aspects of previous versions and covered many components that are important in the evaluation of the driving styles.

## 4. Discussion

The analyses and the obtained results led to the development of a reliable and valid instrument for assessing driving styles in Bulgaria. The final version of the Bulgarian adaptation of the MDSI consists of 57 items measuring eight driving styles—risky, irrational, anxious, high velocity, dissociative, patient and careful, angry and distress reduction driving styles. The consistency of the sums for the whole questionnaire is very high (α = 0.78). Combining items from different versions of the inventory led to its enrichment. The factorial structure of the MDSI-BG is comparable to the previous versions. Six out of the eight MDSI-BG factors are identical to the factorial composition of the MDSI-RO and to the MDSI-S. The original version of the MDSI also obtained an eight-factor structure. There are two main differences between the original version of the MDSI and its Bulgarian version which are as follows: (a) in the original version the patient and the careful driving styles are presented as two different factors, while in the Bulgarian version these two styles formed one factor; (b) the irrational driving style was developed and added to the Romanian version of the MDSI where it was represented by a separate factor and after its implementation to the Bulgarian version this style emerged in a separate factor too.

## 5. Limitations of the Study

Although the results of the study show that the MDSI-BG is a valid and reliable instrument for the assessment of driving style, there are some limitations of the study that should be acknowledged. First, the sample size is not big enough, so it is not representative of the entire country. Second, the research design can be optimised in order to receive more objective data and thus to validate or not the self-reported answers of the participants. Third, the sampling methodology can be further optimised. These limitations should be addressed in future studies.

## 6. Conclusions

For the last two decades, the researchers in the field strive to isolate certain dangerous driving styles by studying drivers and their habits and searching for a connection between these habits and the risky driving behaviour.

The present study will result in a few direct benefits: (a) The adaptation of the MDSI in Bulgaria will provide an opportunity to study Bulgarian drivers‘ behaviour, as it is known that the driving styles may vary between cultures as they are influenced by cultural and contextual factors [20,21]; (b) A contemporary scientific research in the area will raise new questions and will provide a different point of view in this still problematic field; (c) The adaptation of the inventory will serve as a good base upon which other scientists can continue to study the association of driving style with other significant elements of the driving process.

## Figures and Tables

**Table 1:** Confirmatory factor analyses of the multidimensional driving style inventory (MDSI-BG).

Items of MDSI-BG	Factor Loadings							
	1	2	3	4	5	6	7	8
RISKY DRIVING STYLE								
Enjoy the excitement of dangerous driving	0.757							
Drive faster when a vehicle is trying to pass me	0.336							
Enjoy the sensation of driving on the limit	0.817							
Like to take risks while driving	0.765							
Like the thrill of flirting with death or disaster	0.764							
Fix my hair/makeup while driving	*0.326*							
Enjoy the power of the engine	0.652							
Feel the car asking for more speed	0.630							
Enjoy shifting gears quickly	0.564							
IRRATIONAL DRIVING STYLE								
Drive over the speed limit in the city		0.666						
Exceed the 50 km/h speed limit in villages on perfectly straight roads with no obstacles limiting my visibility		0.672						
Exceed 90 km/h speed limit outside towns on perfectly straight roads with no obstacles limiting my visibility		0.658						
Leave the car parked for short periods of 10–15 min in unauthorized places where I think it wouldn‘t create any traffic danger		0.408						
Don‘t fasten my seatbelt during short trips of less than 10–15 min in the city		0.274						
I overtake slower vehicles by crossing the continuous white line when the visibility is very good and there are no other obstacles		0.351						
ANXIOUS DRIVING STYLE								
Feel nervous while driving			0.420					
Feel distressed while driving			0.848					
Driving makes me feel frustrated			0.828					
It worries me when driving in bad weather			0.737					
On a clear freeway, I usually drive at or a little below the speed limit			0.251					
Feel I have control over driving [−]			0.543					
Feel comfortable while driving [−]			0.821					
HIGH VELOCITY DRIVING STYLE								
In a traffic jam, I think about ways to get through the traffic faster				0.592				
When in a traffic jam and the lane next to me starts to move, I try to move into that lane as soon as possible				0.633				
Drive through traffic lights that have just turned red				0.506				
When a traffic light turns green and the car in front of me doesn’t get going immediately, I try to urge the driver to move on				0.729				
Purposely tailgate other drivers				0.382				
Get impatient during rush hours				0.431				
DISSOCIATIVE DRIVING STYLE								
Misjudge the speed of an oncoming vehicle when passing					0.395			
Taking a roundabout path to reach destination					0.508			
Running a red light for going along traffic					0.490			
Intend to switch on the windscreen wipers, but switch on the lights instead					0.632			
Forget that my lights are on full beam until flashed by another motorist					0.562			
Nearly hit something due to misjudging my gap in a parking lot					0.350			
Plan my route badly, so that I hit traffic that I could have avoided					0.596			
Attempt to drive away from traffic lights in third gear					0.569			
Lost in thoughts or distracted, I fail to notice someone at the pedestrian crossings					0.520			
I daydream to pass the time while driving					0.242			
Driving somewhere else to other than the intended destination					0.620			
CAREFUL AND PATIENT DRIVING STYLE								
Tend to drive cautiously						0.594		
Drive cautiously						0.585		
Always ready to react to unexpected makeovers by other drivers						0.265		
Distracted or preoccupied, and suddenly realize the vehicle ahead has slowed down, and have to slam on the breaks to avoid a collision [−]						0.582		
Get a thrill out of breaking the law [−]						0.630		
At an intersection where I have to give right-of-way to oncoming traffic, I wait patiently for cross-traffic to pass						0.425		
Base my behaviour on the motto “better safe than sorry”						0.515		
When a traffic light turns green and the car in front of me doesn’t get going, I just wait for a while until it moves						0.685		
Plan long journeys in advance						0.412		
Wait patiently when you cannot advance the traffic						0.625		
Wait patiently when not having right of way						0.466		
ANGRY DRIVING STYLE								
Swear at other drivers							0.317	
Blow my horn when someone on the road annoys me							0.539	
Flash the car in front of me when the driver annoys me							0.405	
When someone does something on the road that annoys me, I flash them with the high beam							0.405	
When someone tries to skirt in front of me on the road, I drive in an assertive way in order to prevent it							0.656	
Arguing with other drivers or pedestrians							0.511	
Get angry with people driving slow in the fast lane							0.541	
DISTRESS REDUCTION DRIVING STYLE								
Use muscle relaxation techniques while driving								0.766
While driving, I try to relax myself								0.636
Do relaxing activities while driving								0.746
Meditate while driving								0.490
Listen to music to relax while driving								0.550
Enjoy the landscape while driving								0.438

**Table 2:** Summary statistics for the MDSI-BG.

Scales	Number of Items	Cronbach’s Alpha	Min	Max	Mean	SD	Skewness	Kurtosis
Risky style	8	0.863	8.00	43.00	15.76	6.96	1.264	1.708
Irrational style	5	0.805	5.00	28.00	14.15	5.12	0.263	−0.495
Anxious style	6	0.835	6.00	33.00	14.49	5.31	0.739	0.219
High velocity style	6	0.741	6.00	28.00	14.75	4.48	0.386	−0.133
Dissociative style	9	0.767	9.00	43.00	17.36	4.47	1.172	3.991
Patient and Careful	10	0.734	32.00	60.00	48.03	6.34	−0.083	−0.725
Angry style	7	0.746	7.00	30.00	16.07	4.85	0.394	−0.243
Distress reduction	6	0.626	6.00	34.00	16.38	4.58	0.593	1.344
Valid N	456							
